# Neuropsychological, plasma marker, and functional connectivity changes in Alzheimer’s disease patients infected with COVID-19

**DOI:** 10.3389/fnagi.2023.1302281

**Published:** 2023-12-18

**Authors:** Shouzi Zhang, Li Zhang, Li Ma, Haiyan Wu, Lixin Liu, Xuelin He, Maolong Gao, Rui Li

**Affiliations:** ^1^Department of Psychiatry, Beijing Geriatric Hospital, Beijing, China; ^2^Department of Science and Technology, Beijing Geriatric Hospital, Beijing, China; ^3^CAS Key Laboratory of Mental Health, Institute of Psychology, Beijing, China; ^4^Department of Psychology, University of Chinese Academy of Sciences, Beijing, China

**Keywords:** Alzheimer’s disease, COVID-19, P-tau 181, Aβ42, functional connectivity

## Abstract

**Introduction:**

Patients with COVID-19 may experience various neurological conditions, including cognitive impairment, encephalitis, and stroke. This is particularly significant in individuals who already have Alzheimer’s disease (AD), as the cognitive impairments can be more pronounced in these cases. However, the extent and underlying mechanisms of cognitive impairments in COVID-19-infected AD patients have yet to be fully investigated through clinical and neurophysiological approaches.

**Methods:**

This study included a total of 77 AD patients. Cognitive functions were assessed using neuropsychiatric scales for all participants, and plasma biomarkers of amyloid protein and tau protein were measured in a subset of 25 participants. To investigate the changes in functional brain connectivity induced by COVID-19 infection, a cross-sectional neuroimaging design was conducted involving a subset of 37 AD patients, including a control group of 18 AD participants without COVID-19 infection and a COVID-19 group consisting of 19 AD participants.

**Results:**

For the 77 AD patients between the stages of pre and post COVID-19 infection, there were significant differences in cognitive function and psychobehavioral symptoms on the Montreal Scale (MoCA), the neuropsychiatric inventory (NPI), the clinician’s global impression of change (CIBIC-Plus), and the activity of daily living scale (ADL). The COVID-19 infection significantly decreased the plasma biomarker level of Aβ42 and increased the plasma p-tau181 level in AD patients. The COVID-19-infected AD patients show decreased local coherence (LCOR) in the anterior middle temporal gyrus and decreased global correlation (GCOR) in the precuneus and the medial prefrontal cortex.

**Conclusion:**

The findings suggest clinical, cognitive, and neural alterations following COVID-19 infection in AD patients and emphasize the need for close monitoring of symptoms in AD patients who have had COVID-19 and further exploration of the underlying mechanisms.

## 1 Introduction

Alzheimer’s disease (AD) is a neurodegenerative disorder characterized by cognitive impairment and the presence of behavioral and psychological symptoms (BPSD) (Cerejeira et al., 2012; Devshi et al., 2015). The development of AD is influenced by various factors, including stroke and infection (Lee et al., 2021). Notably, during the COVID-19 epidemic, the infection emerged as a significant contributor to neurological damage, potentially increasing the risk of AD development (Chen et al., 2022; Douaud et al., 2022). There are reports indicating that the Severe Acute Respiratory Syndrome Coronavirus 2 (SARS-CoV-2) is a virus with the ability to affect the nervous system (Wang et al., 2021). Apart from respiratory, cardiovascular, and kidney-related symptoms, approximately one-third of COVID-19 patients have also experienced neurological symptoms like confusion, headaches, loss of taste or smell, and enduring neurological issues (Miners et al., 2020; Ciaccio et al., 2021). SARS-CoV-2 is considered an opportunistic pathogen of the central nervous system (CNS), capable of direct invasion, leading to subsequent interactions between the SARS-CoV-2 spike protein and angiotensin-converting enzyme 2 (ACE-2) (Berger, 2020; Dong et al., 2020; Lechien et al., 2020). Within the brain, ACE-2 is found on neurons and glial cells, with an excessive expression noted in the temporal lobe and hippocampus—cerebral regions associated with the development of AD (Dong et al., 2020).

A large number of patients with post-acute sequelae of SARS-CoV-2 face the impact of cognitive complaints commonly referred to as ‘brain fog’ as well as fatigue (Asadi-Pooya et al., 2022). A recent meta-analysis of 81 studies reported that 22% of patients suffered cognitive impairment and 32% experienced fatigue for a duration of 12 weeks or more following their diagnosis of COVID-19 (Tabacof et al., 2022). However, whether COVID-19 may contribute to the development of AD is less known. Over 20 different risk factors such as age, genes, obesity, diabetes, traumatic brain injury, and infection are associated with AD pathogenesis (Armstrong, 2019; Lee et al., 2021). The β-amyloid (Aβ) immunoreactive senile plaques (SP) and tau immunoreactive neurofibrillary tangles (NFT) are the main pathologies of AD (Knopman et al., 2021). The amyloid cascade hypothesis posits that neurodegeneration in AD is a consequence of the abnormal accumulation of Aβ plaques in diverse brain regions, and the Aβ pathway plays a central role in the pathophysiology of AD (Armstrong, 2019; Knopman et al., 2021). Recently, there have been advancements in the detection technology for fluid and PET biomarkers of Aβ pathology from plasma, which has improved the diagnosis of AD (Karikari et al., 2021). Biomarkers indicating the presence of amyloid and tau proteins are essential evidence for diagnosing AD (Janelidze et al., 2016; Jack et al., 2018). Furthermore, recent evidence shows that SARS-CoV-2 is associated with changes in brain structures including olfactory- and memory-related medial temporal lobe areas, raising the possibility that the consequence of the infection contributes to AD development (Douaud et al., 2022). Resting-state functional magnetic resonance imaging (rs-fMRI) has become widely utilized in AD clinical practice due to its user-friendly nature, particularly in bypassing the challenge of assigning tasks to patients (Amemiya et al., 2023). Research has consistently shown that disruptions in the default-mode network (DMN) serve as an early marker of AD (Hojjati et al., 2017; Kvavilashvili et al., 2020). It has recently been suggested that cognitive psychological changes during the COVID-19 pandemic are closely linked to alterations in the DMN (Dubey et al., 2022). It would be intriguing to investigate whether COVID-19 infection targets brain regions critical to AD pathology. In this study based on our clinical cohort, we investigate the influence of COVID-19 infection on cognitive performance and BPSD, plasma biomarkers, and functional brain connectivity in AD patients.

## 2 Methods

### 2.1 Study design and participant cohort

A combination of randomized prospective cohort and retrospective research was conducted at the Clinic and Department of Psychiatry in Beijing Geriatric Hospital (BGH). The study included a total of 77 AD patients from BGH outpatient and ward settings between October 2022 and May 2023. The enrolled participants consisted of mild (*N* = 28), moderate (*N* = 20), and severe (*N* = 29) AD patients, who were 60–96 years old, meeting the National Institute on Aging-Alzheimer’s Association (NIA-AA) AD criteria, and were infected with symptomatic COVID-19 mainly around October to November 2022 at the outpatient clinic and the Department of Psychiatry in BGH. Confirmation of COVID-19 infection was done through a positive real-time reverse transcriptase polymerase chain reaction (RT-PCR) test from a nasopharyngeal and/or throat swab. All COVID-19 infection patients had varying clinical symptoms, such as fever, cough, expectoration, and anorexia. Of all the infected patients, 21 were classified as having severe COVID-19 infection, with chest CT showing obvious manifestations of Viral pneumonia and Bacterial pneumonia, 18 patients required mechanical ventilation, and 5 patients died of severe infection and abnormal coagulation function. Neuropsychological performance was assessed before COVID-19 infection for all 77 AD patients and after infection for the 72 survivors (Table 1). Plasma biomarker assays were performed for a subset of 25 participants both before and after COVID-19 infection (Table 2). To investigate the functional brain connectivity changes induced by COVID-19 infection, a cross-sectional design of neuroimaging was conducted for a subset of 37 AD patients, including a control group of 18 participants who had undergone rs-fMRI before COVID-19 infection and a COVID-19 group of 19 participants within 30 days after symptomatic COVID-19 infection, avoiding the acute phase of the illness. Both groups were comparable in sex, age, and neuropsychological performance (Table 3).

**Table 1:** Participant characteristics and clinical outcomes.

Characteristics	Pre COVID-19 infection	Post COVID-19 infection	Wilcoxon Z	*p*
N (male/female)	77 (30/47)	72 (27/45)	/	/
Age, mean (SD)	82.0 (7.22)	80.0 (6.45)	/	/
Death numbers	/	5	/	/
MMSE, mean (SD)	11.26 (8.63)	9.58 (8.13)	5.916	<0.001
MOCA, mean (SD)	6.28 (6.21)	5.33 (5.65)	5.527	<0.001
NPI, mean (SD)	16.58 (10.61)	17.83 (11.0)	4.901	<0.001
CIBIC-plus, mean (SD)	20.30 (11.75)	22.74 (12.60)	5.865	<0.001
ADL, mean (SD)	54.80 (38.42)	52.71 (38.49)	4.896	<0.001

**Table 2:** Characteristics for plasma biomarker assays.

Characteristics	Pre COVID-19 infection	Post COVID-19 infection	Statistics	*P*
N (male/female)	25 (12/13)	25 (12/13)	/	/
Age, mean (SD)	85.0 (5.268)	85.0 (5.268)	/	/
Aβ42, mean (SD)	18.42 (14.60)	7.39 (2.03)	3.928^a^	<0.001
Aβ40, mean (SD)	115.32 (96.53)	100.79 (25.15)	0.783 ^a^	0.43
Aβ42/Aβ40 ratio, mean (SD)	0.17 (0.12)	0.07 (0.01)	4.084 ^a^	<0.001
p-tau 181, mean (SD)	5.06 (1.52)	5.96 (2.28)	2.464 ^a^	0.005
MMSE, mean (SD)	3.87 (8.12)	3.35 (5.74)	2.232 ^b^	0.026
MOCA, mean (SD)	1.52 (2.81)	1.0 (1.85)	2.232 ^b^	0.026
NPI, mean (SD)	22.79 (10.34)	23.95 (10.01)	2.843 ^b^	0.004
CIBIC-plus, mean (SD)	29.0 (7.83)	31.73 (7.36)	3.296 ^b^	0.001
ADL, mean (SD)	21.30 (19.78)	18.64 (18.72)	2.414 ^b^	0.016

a, two-sample T value; b, Wilcoxon signed rank Z score.

**Table 3:** Characteristics of rs-fMRI participants.

Characteristics	COVID-19	Control	Statistics	*p*
N (male/female)^a^	19 (8/11)	18 (8/10)	0.02^d^	0.89
Age, mean (SD)^b^	84.3 (6.5)	86.1 (4.5)	0.94^e^	0.35
MMSE, mean (SD)^c^	4.4 (7.6)	4.3 (4.7)	1.34^f^	0.22
MOCA, mean (SD)^c^	2.1 (4.0)	2.1 (2.8)	0.81^f^	0.49
ADL, mean (SD)^c^	24.5 (34.5)	24.7 (23.0)	1.63^f^	0.11
NPI, mean (SD)^c^	23.4 (12.9)	22.7 (9.0)	0.54^f^	0.59
CIBIC-Plus, mean (SD)^c^	28.9 (11.3)	30.1 (6.1)	0.56^f^	0.59

a, Chi-square test; b, two-sample t-test; c, Mann–Whitney U test; d, Chi-square score; e, T value; f, Z value.

### 2.2 Neuropsychological assessment

The battery of cognitive and psychiatric scales was administered to enrolled participants by five neurologists at the Beijing Geriatric Hospital prior to COVID-19 infection. The assessments after COVID-19 infection were performed at a single time point, avoiding the acute phase of the illness and within 30 days after infection. These scales included cognitive assessment (mini-mental state examination, MMSE; Montreal Cognitive Assessment, MoCA); psychiatric assessment (Neuropsychiatric inventory, NPI), functional ability and quality of life assessment (Activities of Daily Living, ADL), and comprehensive assessment (Clinician’s Interview-Based Impression of Change plus caregiver input, CIBIC-Plus).

### 2.3 Neurobiomarker assays

The plasma samples were collected in polypropylene tubes before COVID-19 infection for the enrolled participants in outpatient and psychiatric wards in Beijing Geriatric Hospital, with immediate low-temperature transportation. A plasma sample was extracted for every participant within 30 days after COVID-19 infection, avoiding the acute phase of the illness. Plasma biomarker assays were conducted at the Beijing Jinyu Medical Neurochemistry Laboratory with the Simoa Human Neurology HD-X (Quanterix). Aβ42, Aβ40, and P-tau 181 were measured, and the Aβ42/Aβ40 ratio was calculated and used as a marker of amyloid accumulation (Hansson et al., 2019). Serum Aβ42 (human Aβ-42 test kit. no. LD106019), Serum Aβ40 (human Aβ-40 test kit. no. LD106007), and P-tau 181 (P-tau 181 V2.1 test kit.no.10411) were measured by single-molecule array (Simoa). The manufacturer’s recommendations for handling and analyzing serum samples were followed, with each Simoa kit’s run including an 8-point calibration curve for each marker and two internal controls.

### 2.4 Image acquisition

Neuroimaging data was collected using a PHILIPS 3-Tesla magnetic resonance imaging system at the BGH. The participants were instructed to keep their eyes closed, not to think of anything in particular, and to lie quietly. Functional images were collected using the parameters: time repetition (TR), 3,000 ms; time echo (TE), 35 ms; field of view (FOV), 240 mm × 240 mm; voxel size, 2 mm × 2 mm; thickness, 4.0 mm; 40 axial slices; matrix, 120 × 116. A high-resolution T1-weighted structural image was acquired for each participant, using the parameters: TR, 7.9 ms; TE, 3.5 ms; 180 slices; acquisition matrix, 160 × 211; voxel size, 1 mm × 1 mm × 1 mm.

### 2.5 Neuroimaging data analysis

#### 2.5.1 Preprocessing

We used the CONN20b toolbox (Nieto-Castanon, 2020) to preprocess and analyze the imaging data. The default preprocessing pipeline for volume-based analyzes, including realignment, slice-timing correction, outlier identification, normalization to Montreal Neurological Institute (MNI) space (3 mm3), and smoothing with a Gaussian kernel of 8 mm full-width half-maximum was performed. The anatomical component-based noise correction (aCompCor) procedure was used to exclude several confounding effects, including noise from the white matter and cerebrospinal areas, 24 parameters of head motions, scrubbing parameters of outlier scans, and linear signal trends. Finally, temporal band-pass filtering (0.01–0.08 Hz) was performed to reduce the effects of low-frequency drift and physiological high-frequency noise.

#### 2.5.2 Functional connectivity measures

We used the voxel-wise network measure of global correlation (GCOR) and local correlation (LCOR) to summarize the properties of the whole-brain voxel-to-voxel connectome, i.e., all the functional connections between each pair of voxels in the brain (Nieto-Castanon, 2020). GCOR measures centrality at each voxel, characterized by the strength and sign of connectivity between a given voxel and the rest of the brain (Nieto-Castanon, 2020). GCOR is defined as the average of correlation coefficients between each individual voxel and all of the voxels in the brain (Saad et al., 2013). LCOR measures local coherence at each voxel, characterized by the strength and sign of connectivity between a given voxel and the neighboring voxels in the brain (Nieto-Castanon, 2020). LCOR is defined as the average of correlation coefficients between each individual voxel and a region of neighboring voxels (Deshpande et al., 2009).

### 2.6 Statistical analyzes

The statistical analyzes of neuropsychological and biomarker data were conducted using SPSS 21.0 (IBM Corp. in Armonk, NY, United States). The normality of the data was first examined using the Shapiro–Wilk test. Subsequently, paired comparisons of neuropsychological performance before and after COVID-19 infection on MMSE, MoCA, ADL, and CIBIC-Plus were assessed using the Wilcoxon signed-rank test (*p* < 0.05). For the comparison of biomarkers (Aβ42, Aβ40, and p-tau 181), a paired t-test was employed (*p* < 0.05). Additionally, the Mann–Whitney U test was used to compare group differences in neuropsychological performance within the rs-fMRI subset (*p* < 0.05). The Chi-square test was utilized for sex distribution (*p* < 0.05). Functional connectivity measures of LCOR and GCOR in the COVID-19 group and control group were compared using the two-sample t-test in CONN toolbox with age and sex as covariates. The relationship between functional connectivity measure and neuropsychological data was examined using Spearman correlation (*p* < 0.05).

## 3 Results

### 3.1 Neuropsychological performance

The results of the Wilcoxon signed-rank test indicate that COVID-19 induces systematic changes in all five measured neuropsychological tests (Table 1). Patients infected with COVID-19 exhibited a significant decrease in cognitive performance on both MMSE and MoCA, as well as worse psychiatric performance on NPI, daily performance on ADL, and comprehensive performance on CIBIC-Plus (all *p*s < 0.001). By analyzing these scales, the deteriorated domains of memory (97%), language (67%), executive function (70%), somnipathy (75%), apathy (65%), anxiety (42%), depression (33%), and anorexia (7%) were found in AD patients infected with COVID-19. The comparison of neuropsychological performance changes in AD patients with severe COVID-19 infection and those with not-severe infection using the Mann–Whitney U test revealed that AD patients with severe COVID-19 infection exhibited more rapid clinical changes in ADL (Z = 2.377, *p* = 0.017), NPI (Z = 2.141, *p* = 0.032) and CIBIC-Plus (Z = 2.045, *p* = 0.041).

### 3.2 Plasma biomarkers

**Figure 1:**
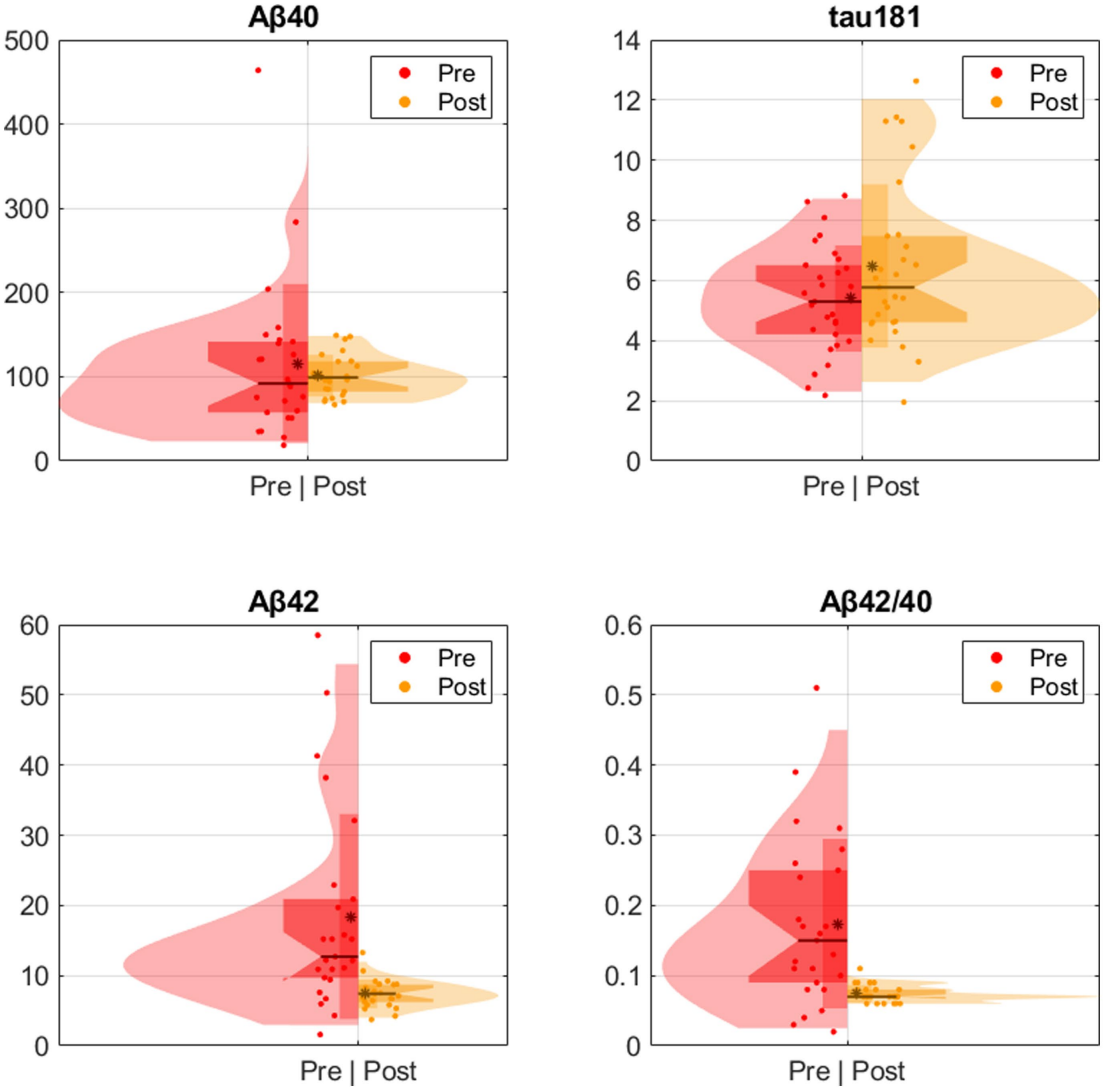
Pre and post COVID-19 infection participant plasma Aβ42, Aβ40, Aβ42/Aβ40 and P-tau 181.

A paired t-test on a subset of 25 participants revealed that COVID-19 induced an increase in the levels of P-tau 181 (*p* = 0.005), a decrease in the levels of Aβ42 (*p* < 0.001), and a reduction in the Aβ42/Aβ40 ratio (*p* < 0.001). No significant difference was found in the levels of Aβ40 (*p* = 0.43) (Table 2 and Figure 1).

### 3.3 Functional connectivity

**Figure 2:**
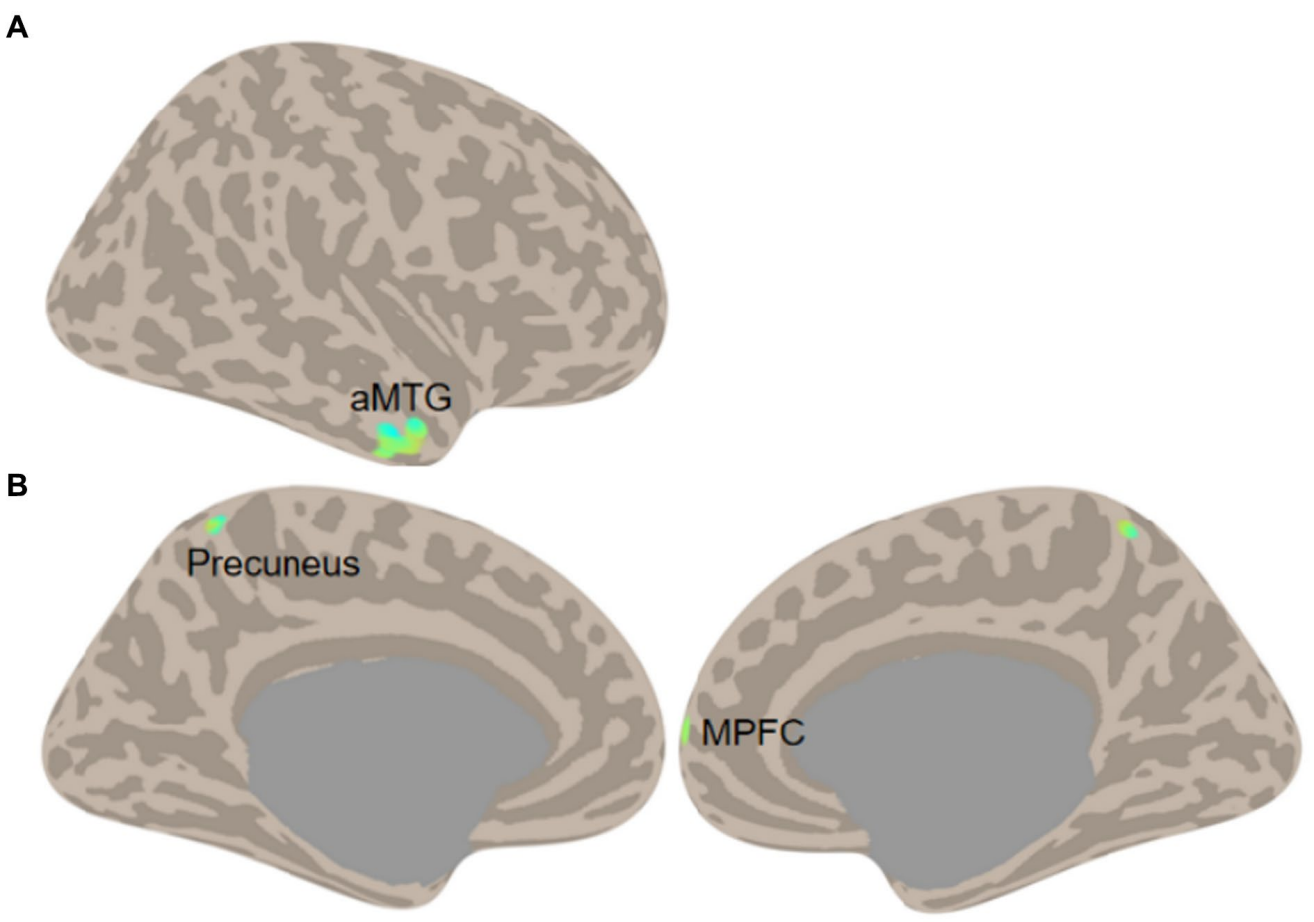
Functional connectivity comparisons (**A**, LCOR; **B**, GCOR) between COVID-19 cases and controls in AD (Two-sample *t*-test of COVID-19 vs. Control, threshold at voxel *p* < 0.001 and cluster size *k* > 20 voxels).

A two-sample t-test was conducted on LCOR and GCOR, which revealed no significant difference between the COVID-19-infected group and the control group under the false discovery rate (FDR) corrected significance level of *p* < 0.05. The uncorrected results, using a significance level of *p* < 0.001 and a cluster size of k > 20 voxels, were thus reported. As expected, changes in functional connectivity were observed in regions that have been previously linked to AD pathology. Specifically, AD patients infected with COVID-19 showed decreased LCOR in the anterior middle temporal gyrus (aMTG; 52, 6, −30; 77 voxels) and decreased GCOR in the precuneus (−2, −54, 66; 30 voxels) and the medial prefrontal cortex (MPFC; 28, 66, 2; 22 voxels) (Figure 2). However, no significant correlation was found between functional connectivity in these regions and neuropsychological data (all *p*s > 0.05).

## 4 Discussion

We explored the neurocognitive and psychiatric outcomes at a one-month follow-up in AD patients who had contracted COVID-19. It is well-documented that neurocognitive impairment is frequently observed in individuals without prior cognitive deficits following COVID-19 infection. Additionally, psychiatric symptoms such as persistent anxiety, insomnia, and depression have been commonly reported. Previous studies have indicated that these sequelae are particularly pronounced in patients with a history of psychiatric disorders (Gennaro et al., 2021). Our study expanded the investigation into the impact of COVID-19 on AD patients. We observed a broad spectrum of cognitive decline across memory, language, and executive function domains in AD patients following COVID-19 infection. Additionally, prominent behavioral and psychological manifestations included apathy, anxiety, depression, and sleep disturbances. Our study involved a one-month follow-up to assess clinical cognitive and psychiatric impairment. It is worth noting that in several previous studies, these neurological sequelae have been observed to persist for at least 3 months. These sequelae present in various forms, encompassing issues related to the sense of smell, stroke, encephalopathy, Guillain–Barre syndrome (GBS), neurological inflammation (such as myelitis, encephalitis, and meningitis), seizures, cognitive impairment, muscle pain, headaches, dizziness, and fatigue. Furthermore, they may encompass neuropsychiatric symptoms such as psychosis, anxiety, depression, and sleep disorders (Divani et al., 2020; Collantes et al., 2021; Roy et al., 2021; Yassin et al., 2021). There is not always a direct correlation between the severity of COVID-19 infection and the presence of neurological symptoms (Rogers et al., 2021).

The various mechanisms or the timing of neurological injuries may be associated with SARS-CoV-2 infection. We have observed significant changes in plasma P-tau 181 and Aβ levels before and after COVID-19 infection, consistent with the study by Frontera et al. (2022). Their research team discovered notable increases in serum T-tau, P-tau 181, glial fibrillary acidic protein (GFAP), and neurofilament light chain (NfL) levels in individuals with COVID-19-induced encephalopathy. Moreover, these markers showed associations with the severity of the disease. COVID-19 patients demonstrated higher levels of NfL and GFAP in comparison to non-COVID controls with mild cognitive impairment (MCI) or AD (Frontera et al., 2022). Ziff et al. (2022) conducted a study in which they measured CSF amyloid precursor protein soluble (sAPPs), NfL, P-tau, Aβ42, and Aβ40 in 21 COVID-19 neurological cases and 23 non-COVID controls. They found that the levels of Aβ42, Aβ40, and sAPPs were reduced in COVID-19 neurological cases compared to controls. These changes were associated with increased levels of neuroinflammatory markers such as CSF proinflammatory cytokines (TNFɑ, IL6, IL1β, IL8) and NfL (Ziff et al., 2022). Neuropathological data from COVID-19 decedents have shown evidence of endothelial inflammation, hypoxic injury, and disruption of the blood–brain barrier (BBB) (Hanley et al., 2020; Kirschenbaum et al., 2021; Pajo et al., 2021). Elevated inflammatory markers in COVID-19 infection, including IL-6, D-dimer, CRP, and ferritin, have been shown to correlate with cognitive dysfunction and the promotion of amyloid plaque and neurofibrillary tangle pathology in animal models (Shen et al., 2019; Frontera et al., 2021). It is intriguing to note that hypoxia can induce tau phosphorylation, which is a core pathology correlated with corresponding memory deficits in AD (Zhang et al., 2014). Additionally, several studies have demonstrated elevated levels of T-tau, NfL, and GFAP in the serum and cerebrospinal fluid (CSF) of COVID-19 patients compared to normal controls, with none of these studies explicitly excluding COVID-19 infection in individuals with a baseline cognitive impairment or dementia history (Ameres et al., 2020; De Lorenzo et al., 2021; Eden et al., 2021). This study appears to be the first to specifically target the AD population affected by COVID-19 infection and has confirmed the elevation of plasma P-tau181 and Aβ42. This underscores the importance of understanding the potential impact of COVID-19 on individuals with underlying neurodegenerative conditions.

There is indeed speculation among neuroscientists that SARS-CoV-2 might exacerbate cognitive decline in Alzheimer’s disease (AD) through neurotoxicity or by triggering the host immune response. This could potentially lead to demyelination, neurodegeneration, and cellular senescence (Ciaccio et al., 2021). This hypothesis underscores the importance of understanding the potential interactions between COVID-19 and neurodegenerative conditions, such as AD, and the need for further research in this area. ACE-2 expression plays a crucial role in viral tropism and the pathogenesis of COVID-19. Post-mortem studies have indicated increased ACE-2 expression in the brains of AD patients compared to control subjects (Ding et al., 2020). The increased prevalence of SARS-CoV-2 infection in individuals with AD may be attributed to shared risk factors and comorbidities between AD and COVID-19. These commonalities include factors like advanced age, gender, APOE Ɛ4 expression, and elevated ACE-2 expression in the AD brain. Moreover, SARS-CoV-2 can directly impact the central nervous system by using retrograde axonal transport through olfactory and enteric neurons, infecting lymphocytes, and compromising the BBB integrity. Notably, the elderly population may be more susceptible to neuroinvasion during SARS-CoV-2 infection due to the gradual loss of BBB integrity (Montagne et al., 2015; Hascup and Hascup, 2020).

The cross-sectional comparison further reveals that COVID-19 infection in AD patients is associated with decreased functional brain connectivity. Specifically, there was a decline in local coherence in the aMTG and global correlation in the precuneus and MPFC. The aMTG is known for its critical involvement in memory (Jang et al., 2017; Fassbender et al., 2022) and language (Binder et al., 2011; Sugimoto et al., 2023), and it is associated with AD biomarkers (Jiang et al., 2016). The precuneus and MPFC are crucial regions of the default-mode network and serve as hubs of the whole brain (Liang et al., 2013). Dysfunction within the DMN has been consistently observed in AD patients and has been linked to memory impairment and AD biomarkers (Menon, 2023). Recent findings have suggested that cognitive decline in AD is reflected by a decrease in signal complexity within DMN nodes (Grieder et al., 2018), which may explain the overall decline in connectivity observed in DMN regions throughout the brain. The convergence of COVID-19 effects and AD pathology in the brain may suggest that COVID-19 could potentially accelerate the progression of the disease.

Several limitations must be pointed out. First, the observed clinical changes suggest an increase in AD progression in COVID-19-infected patients. For instance, previous longitudinal research has consistently shown an annual MMSE decline of 2 to 3 points in AD patients (Clark et al., 1999; Scharre et al., 2021). Our data reveals that the average MMSE score in AD patients infected with COVID-19 decreased by 2.01 points over approximately 1 month, a change that mirrors the annual decline attributed to the natural evolution of AD. However, the self-controlled design used to study the influence of COVID-19 infection on neuropsychological performance and plasma markers in AD patients presents challenges in disentangling the impact of normal disease progression from that of COVID-19 infection, as well as understanding their potential interactions. It should be noted that although the decline observed in our study is larger than what would be expected in a normal cohort of AD patients, we cannot conclusively attribute this effect entirely to COVID-19. Our study lacked a control group, and other confounding factors, such as hospitalization and the use of medications, may have influenced the results. To more accurately determine the effect of COVID-19 on these markers in AD patients, real cohort studies with appropriate controls must be conducted. Second, in terms of the functional connectivity changes observed, it is important to note that while we did match the cognitive performance of patients across both groups, a longitudinal design would be necessary to validate whether these changes actually predict a heightened rate of cognitive decline in COVID-19-infected patients. Additionally, we would like to highlight that these functional connectivity changes were not corrected for multiple comparisons, and thus, we must acknowledge that there may be a higher false positive rate in the results. Third, it is important to note that the sample size in both the plasma biomarker subset and the rs-fMRI subset is small, and the clinical and disease information of the participants in our study was incomplete, lacking information on the duration of AD and COVID-19 infection, medication usage, and health history, for example. These limitations restricted our ability to conduct a more comprehensive mechanistic analysis of the observed changes.

The findings also have significant clinical implications. The observed cognitive impairments, alterations in plasma biomarkers, and changes in functional brain connectivity in COVID-19-infected AD patients underscore the need for targeted clinical management strategies for this specific subgroup. These findings emphasize the importance of closely monitoring symptoms in AD patients who have had COVID-19, as the impact on these individuals appears to be substantial. The alterations in plasma biomarkers and functional brain connectivity highlight the need for further investigations into the underlying mechanisms linking COVID-19 and AD, potentially leading to the development of targeted interventions to mitigate exacerbated cognitive decline and neurological symptoms in this population. Future research directions could include larger-scale studies with more comprehensive clinical and disease information to enable a more in-depth mechanistic analysis of the observed changes, long-term follow-up studies to understand the trajectory of cognitive and neurological changes post COVID-19 infection, and the exploration of potential interventions tailored specifically to AD patients with a history of COVID-19. By addressing these issues, future research could significantly advance our understanding of the interplay between COVID-19 and AD, potentially leading to improved clinical management and care for these patients.

## Data availability statement

The original contributions presented in the study are included in the article/supplementary material, further inquiries can be directed to the corresponding authors.

## Ethics statement

Studies involving human participants were reviewed and approved by the Ethics Committee of Beijing Geriatric Hospital. The patients/participants legal guardian/next of kin provided written informed consent to participate in this study.

## Author contributions

SZ: Methodology, Writing – original draft, Writing – review & editing. LZ: Project administration, Writing – original draft. LM: Project administration, Writing – review & editing. HW: Project administration, Writing – review & editing. LL: Project administration, Writing – review & editing. XH: Project administration, Writing – review & editing. MG: Writing – review & editing, Formal analysis, Methodology. RL: Writing – review & editing, Data curation, Supervision, Writing – original draft.

## References

[ref1] Amemiya S. Takao H. Abe O. (2023). Resting-state fMRI: emerging concepts for future clinical application. J. Magn. Reson. Imaging. doi: 10.1002/jmri.28894, PMID: 37424140

[ref2] Ameres M. Brandstetter S. Toncheva A. A. Kabesch M. Leppert D. Kuhle J.. (2020). Association of neuronal injury blood marker neurofilament light chain with mild-to-moderate COVID-19. J. Neurol. 267, 3476–3478. doi: 10.1007/s00415-020-10050-y, PMID: 32647900 PMC7345451

[ref3] Armstrong R. A. (2019). Risk factors for Alzheimer's disease. Folia Neuropathol. 57, 87–105. doi: 10.5114/fn.2019.85929, PMID: 31556570

[ref4] Asadi-Pooya A. A. Akbari A. Emami A. Lotfi M. Rostamihosseinkhani M. Nemati H.. (2022). Long COVID syndrome-associated brain fog. J. Med. Virol. 94, 979–984. doi: 10.1002/jmv.27404, PMID: 34672377 PMC8662118

[ref5] Berger J. R. (2020). COVID-19 and the nervous system. J. Neurovirol. 26, 143–148. doi: 10.1007/s13365-020-00840-5, PMID: 32447630 PMC7245181

[ref6] Binder J. R. Gross W. L. Allendorfer J. B. Bonilha L. Chapin J. Edwards J. C.. (2011). Mapping anterior temporal lobe language areas with fMRI: a multicenter normative study. NeuroImage 54, 1465–1475. doi: 10.1016/j.neuroimage.2010.09.048, PMID: 20884358 PMC2997157

[ref7] Cerejeira J. Lagarto L. Mukaetova-Ladinska E. B. (2012). Behavioral and psychological symptoms of dementia. Front. Neurol. 3:73. doi: 10.3389/fneur.2012.00073, PMID: 22586419 PMC3345875

[ref8] Chen F. Chen Y. Wang Y. Ke Q. Cui L. (2022). The COVID-19 pandemic and Alzheimer's disease: mutual risks and mechanisms. Transl Neurodegener 11:40. doi: 10.1186/s40035-022-00316-y 36089575 PMC9464468

[ref9] Ciaccio M. Lo Sasso B. Scazzone C. Gambino C. M. Ciaccio A. M. Bivona G.. (2021). COVID-19 and Alzheimer's disease. Brain Sci. 11:305. doi: 10.3390/brainsci11030305, PMID: 33673697 PMC7997244

[ref10] Clark C. M. Sheppard L. Fillenbaum G. G. Galasko D. Morris J. C. Koss E.. (1999). Variability in annual Mini-Mental State Examination score in patients with probable Alzheimer disease: a clinical perspective of data from the Consortium to Establish a Registry for Alzheimer's Disease. Arch. Neurol. 56, 857–862. doi: 10.1001/archneur.56.7.857, PMID: 10404988

[ref11] Collantes M. E. V. Espiritu A. I. Sy M. C. C. Anlacan V. M. M. Jamora R. D. G. (2021). Neurological manifestations in COVID-19 infection: a systematic review and meta-analysis. Can. J. Neurol. Sci. 48, 66–76. doi: 10.1017/cjn.2020.146, PMID: 32665054 PMC7492583

[ref12] De Lorenzo R. Lore N. I. Finardi A. Mandelli A. Cirillo D. M. Tresoldi C.. (2021). Blood neurofilament light chain and total tau levels at admission predict death in COVID-19 patients. J. Neurol. 268, 4436–4442. doi: 10.1007/s00415-021-10595-6, PMID: 33973106 PMC8108733

[ref13] Deshpande G. Laconte S. Peltier S. Hu X. (2009). Integrated local correlation: a new measure of local coherence in fMRI data. Hum. Brain Mapp. 30, 13–23. doi: 10.1002/hbm.20482, PMID: 17979117 PMC6870773

[ref14] Devshi R. Shaw S. Elliott-King J. Hogervorst E. Hiremath A. Velayudhan L.. (2015). Prevalence of behavioural and psychological symptoms of dementia in individuals with learning disabilities. Diagnostics (Basel, Switzerland) 5, 564–576. doi: 10.3390/diagnostics5040564, PMID: 26854171 PMC4728475

[ref15] Ding Q. Shults N. V. Harris B. T. Suzuki Y. J. (2020). Angiotensin-converting enzyme 2 (ACE2) is upregulated in Alzheimer's disease brain. bioRxiv 8:2020.10.08.331157. doi: 10.1101/2020.10.08.331157 PMC7914443 33567524

[ref16] Divani A. A. Andalib S. Biller J. Napoli D. M. Moghimi N. Rubinos C. A.. (2020). Central nervous system manifestations associated with COVID-19. Curr. Neurol. Neurosci. Rep. 20:60. doi: 10.1007/s11910-020-01086-8, PMID: 33128130 PMC7599061

[ref17] Dong M. Zhang J. Ma X. Tan J. Chen L. Liu S.. (2020). ACE2, TMPRSS2 distribution and extrapulmonary organ injury in patients with COVID-19. Biomed. Pharmacother. 131:110678. doi: 10.1016/j.biopha.2020.110678, PMID: 32861070 PMC7444942

[ref18] Douaud G. Lee S. Alfaro-Almagro F. Arthofer C. Wang C. Mccarthy P.. (2022). SARS-CoV-2 is associated with changes in brain structure in UK biobank. Nature 604, 697–707. doi: 10.1038/s41586-022-04569-5, PMID: 35255491 PMC9046077

[ref19] Dubey S. Dubey M. J. Ghosh R. Mitchell A. J. Chatterjee S. Das S.. (2022). The cognitive basis of psychosocial impact in COVID-19 pandemic. Does it encircle the default mode network of the brain? A pragmatic proposal. Med. Res. Arch. 10:10.18103/mra.v10i3.2707. doi: 10.18103/mra.v10i3.2707, PMID: 35530572 PMC9071110

[ref20] Eden A. Kanberg N. Gostner J. Fuchs D. Hagberg L. Andersson L.-M.. (2021). CSF biomarkers in patients with COVID-19 and neurologic symptoms a case series. Neurology 96, E294–E300. doi: 10.1212/WNL.0000000000010977, PMID: 33004602

[ref21] Fassbender R. V. Risius O. J. Dronse J. Richter N. Gramespacher H. Befahr Q.. (2022). Decreased efficiency of between-network dynamics during early memory consolidation with aging. Front. Aging Neurosci. 14:780630. doi: 10.3389/fnagi.2022.780630, PMID: 35651531 PMC9148994

[ref22] Frontera J. A. Boutajangout A. Masurkar A. V. Betensky R. A. Ge Y. Vedvyas A.. (2022). Comparison of serum neurodegenerative biomarkers among hospitalized COVID-19 patients versus non-COVID subjects with normal cognition, mild cognitive impairment, or Alzheimer's dementia. Alzheimers Dement. 18, 899–910. doi: 10.1002/alz.12556, PMID: 35023610 PMC9011610

[ref23] Frontera J. A. Sabadia S. Lalchan R. Fang T. Flusty B. Millar-Vernetti P.. (2021). A prospective study of neurologic disorders in hospitalized patients with COVID-19 in new York City. Neurology 96, E575–E586. doi: 10.1212/WNL.0000000000010979, PMID: 33020166 PMC7905791

[ref24] Gennaro M. M. Mariagrazia P. Rebecca D. L. Cristiano M. Sara P. Roberto F.. (2021). Persistent psychopathology and neurocognitive impairment in COVID-19 survivors: effect of inflammatory biomarkers at three-month follow-up. Brain Behav. Immun. 94, 138–147. doi: 10.1016/j.bbi.2021.02.021, PMID: 33639239 PMC7903920

[ref25] Grieder M. Wang D. J. J. Dierks T. Wahlund L.-O. Jann K. (2018). Default mode network complexity and cognitive decline in mild Alzheimer's disease. Front. Neurosci. 12:770. doi: 10.3389/fnins.2018.00770, PMID: 30405347 PMC6206840

[ref26] Hanley B. Naresh K. N. Roufosse C. Nicholson A. G. Weir J. Cooke G. S.. (2020). Histopathological findings and viral tropism in UK patients with severe fatal COVID-19: a post-mortem study. Lancet Microbe. 1, E245–E253. doi: 10.1016/S2666-5247(20)30115-4, PMID: 32844161 PMC7440861

[ref27] Hansson O. Lehmann S. Otto M. Zetterberg H. Lewczuk P. (2019). Advantages and disadvantages of the use of the CSF amyloid (a) 42/40 ratio in the diagnosis of Alzheimer's disease. Alzheimers Res. Ther. 11:34. doi: 10.1186/s13195-019-0485-0, PMID: 31010420 PMC6477717

[ref28] Hascup E. R. Hascup K. N. (2020). Does SARS-CoV-2 infection cause chronic neurological complications? Geroscience 42, 1083–1087. doi: 10.1007/s11357-020-00207-y, PMID: 32451846 PMC7247778

[ref29] Hojjati S. H. Ebrahimzadeh A. Khazaee A. Babajani-Feremi A. Alzheimer’s Disease Neuroimaging Initiative (2017). Predicting conversion from MCI to AD using resting-state fMRI, graph theoretical approach and SVM. J. Neurosci. Methods 282, 69–80. doi: 10.1016/j.jneumeth.2017.03.006, PMID: 28286064

[ref30] Jack C. R. Bennett D. A. Blennow K. Carrillo M. C. Dunn B. Haeberlein S. B.. (2018). NIA-AA research framework: toward a biological definition of Alzheimer's disease. Alzheimers Dement. 14, 535–562. doi: 10.1016/j.jalz.2018.02.018, PMID: 29653606 PMC5958625

[ref31] Janelidze S. Stomrud E. Palmqvist S. Zetterberg H. Van Westen D. Jeromin A.. (2016). Plasma β-amyloid in Alzheimer's disease and vascular disease. Sci. Rep. 6:26801. doi: 10.1038/srep26801, PMID: 27241045 PMC4886210

[ref32] Jang A. I. Wittig J. H. Inati S. K. Zaghloul K. A. (2017). Human cortical neurons in the anterior temporal lobe reinstate spiking activity during verbal memory retrieval. Curr. Biol. 27:1700-+. doi: 10.1016/j.cub.2017.05.014, PMID: 28552361 PMC5508588

[ref33] Jiang Y. Huang H. Abner E. Broster L. S. Jicha G. A. Schmitt F. A.. (2016). Alzheimer's biomarkers are correlated with brain connectivity in order adults differentially during resting and task states. Front. Aging Neurosci. 8:15. doi: 10.3389/fnagi.2016.00015, PMID: 26903858 PMC4744860

[ref34] Karikari T. K. Benedet A. L. Ashton N. J. Lantero Rodriguez J. Snellman A. Suarez-Calvet M.. (2021). Diagnostic performance and prediction of clinical progression of plasma phospho-tau181 in the Alzheimer's disease Neuroimaging initiative. Mol. Psychiatry 26, 429–442. doi: 10.1038/s41380-020-00923-z, PMID: 33106600

[ref35] Kirschenbaum D. Imbach L. L. Rushing E. J. Frauenknecht K. B. M. Gascho D. Ineichen B. V.. (2021). Intracerebral endotheliitis and microbleeds are neuropathological features of COVID-19. Neuropathol. Appl. Neurobiol. 47, 454–459. doi: 10.1111/nan.12677, PMID: 33249605 PMC7753688

[ref36] Knopman D. S. Amieva H. Petersen R. C. Chetelat G. Holtzman D. M. Hyman B. T.. (2021). Alzheimer disease. Nat. Rev. Dis. Primers. 7:33. doi: 10.1038/s41572-021-00269-y, PMID: 33986301 PMC8574196

[ref37] Kvavilashvili L. Niedzwienska A. Gilbert S. J. Markostamou I. (2020). Deficits in spontaneous cognition as an early marker of Alzheimer's disease. Trends Cogn. Sci. 24, 285–301. doi: 10.1016/j.tics.2020.01.005, PMID: 32160566

[ref38] Lechien J. R. Chiesa-Estomba C. M. De Siati D. R. Horoi M. Le Bon S. D. Rodriguez A.. (2020). Olfactory and gustatory dysfunctions as a clinical presentation of mild-to-moderate forms of the coronavirus disease (COVID-19): a multicenter European study. Eur. Arch. Otorhinolaryngol. 277, 2251–2261. doi: 10.1007/s00405-020-05965-1, PMID: 32253535 PMC7134551

[ref39] Lee J.-H. Lee C. J. Park J. Lee S. J. Choi S.-H. (2021). The Neuroinflammasome in Alzheimer's disease and cerebral stroke. Dement Geriatr. Cogn. Dis. Extra. 11, 159–167. doi: 10.1159/000516074, PMID: 34249072 PMC8255751

[ref40] Liang X. Zou Q. He Y. Yang Y. (2013). Coupling of functional connectivity and regional cerebral blood flow reveals a physiological basis for network hubs of the human brain. Proc. Natl. Acad. Sci. U. S. A. 110, 1929–1934. doi: 10.1073/pnas.1214900110, PMID: 23319644 PMC3562840

[ref41] Menon V. (2023). 20 years of the default mode network: a review and synthesis. Neuron 111, 2469–2487. doi: 10.1016/j.neuron.2023.04.023, PMID: 37167968 PMC10524518

[ref42] Miners S. Kehoe P. G. Love S. (2020). Cognitive impact of COVID-19: looking beyond the short term. Alzheimers Res. Ther. 12:170. doi: 10.1186/s13195-020-00744-w, PMID: 33380345 PMC7772800

[ref43] Montagne A. Barnes S. R. Sweeney M. D. Halliday M. R. Sagare A. P. Zhao Z.. (2015). Blood-brain barrier breakdown in the aging human hippocampus. Neuron 85, 296–302. doi: 10.1016/j.neuron.2014.12.032, PMID: 25611508 PMC4350773

[ref44] Nieto-Castanon A. (2020). Handbook of functional connectivity magnetic resonance imaging methods in CONN. Boston, MA: Hilbert Press.

[ref45] Pajo A. T. Espiritu A. I. Apor A. D. A. O. Jamora R. D. G. (2021). Neuropathologic findings of patients with COVID-19: a systematic review. Neurol. Sci. 42, 1255–1266. doi: 10.1007/s10072-021-05068-7, PMID: 33483885 PMC7822400

[ref46] Rogers J. P. Watson C. J. Badenoch J. Cross B. Butler M. Song J.. (2021). Neurology and neuropsychiatry of COVID-19: a systematic review and meta-analysis of the early literature reveals frequent CNS manifestations and key emerging narratives. J. Neurol. Neurosurg. Psychiatry 92, 932–941. doi: 10.1136/jnnp-2021-326405, PMID: 34083395

[ref47] Roy D. Ghosh R. Dubey S. Dubey M. J. Benito-Leon J. Kanti Ray B. (2021). Neurological and neuropsychiatric impacts of COVID-19 pandemic. Can. J. Neurol. Sci. 48, 9–24. doi: 10.1017/cjn.2020.173, PMID: 32753076 PMC7533477

[ref48] Saad Z. S. Reynolds R. C. Jo H. J. Gotts S. J. Chen G. Martin A.. (2013). Correcting brain-wide correlation differences in resting-state FMRI. Brain Connect. 3, 339–352. doi: 10.1089/brain.2013.0156, PMID: 23705677 PMC3749702

[ref49] Scharre D. W. Chang S. I. Nagaraja H. N. Wheeler N. C. Kataki M. (2021). Self-administered Gerocognitive examination: longitudinal cohort testing for the early detection of dementia conversion. Alzheimers Res. Ther. 13:192. doi: 10.1186/s13195-021-00930-4, PMID: 34872596 PMC8650250

[ref50] Shen X.-N. Niu L.-D. Wang Y.-J. Cao X.-P. Liu Q. Tan L.. (2019). Inflammatory markers in Alzheimer's disease and mild cognitive impairment: a meta-analysis and systematic review of 170 studies. J. Neurol. Neurosurg. Psychiatry 90, 590–598. doi: 10.1136/jnnp-2018-319148, PMID: 30630955

[ref51] Sugimoto H. Abe M. S. Otake-Matsuura M. (2023). Word-producing brain: contribution of the left anterior middle temporal gyrus to word production patterns in spoken language. Brain Lang. 238:105233. doi: 10.1016/j.bandl.2023.105233, PMID: 36842390

[ref52] Tabacof L. Tosto-Mancuso J. Wood J. Cortes M. Kontorovich A. Mccarthy D.. (2022). Post-acute COVID-19 syndrome negatively impacts physical function, cognitive function, health-related quality of life, and participation. Am. J. Phys. Med. Rehabil. 101, 48–52. doi: 10.1097/PHM.0000000000001910, PMID: 34686631 PMC8667685

[ref53] Wang D. Du T. Hong W. Chen L. Que H. Lu S.. (2021). Neurological complications and infection mechanism of SARS-CoV-2. Signal Transduct. Target. Ther. 6, 3534–3549. doi: 10.1038/s41392-021-00818-7 PMC8609271 34815399

[ref54] Yassin A. Nawaiseh M. Shaban A. Alsherbini K. El-Salem K. Soudah O.. (2021). Neurological manifestations and complications of coronavirus disease 2019 (COVID-19): a systematic review and meta-analysis. BMC Neurol. 21:138. doi: 10.1186/s12883-021-02161-4, PMID: 33784985 PMC8007661

[ref55] Zhang C.-E. Yang X. Li L. Sui X. Tian Q. Wei W.. (2014). Hypoxia-induced tau phosphorylation and memory deficit in rats. Neurodegener. Dis. 14, 107–116. doi: 10.1159/000362239, PMID: 24993525

[ref56] Ziff O. J. Ashton N. J. Mehta P. R. Brown R. Athauda D. Heaney J.. (2022). Amyloid processing in COVID-19-associated neurological syndromes. J. Neurochem. 161, 146–157. doi: 10.1111/jnc.15585, PMID: 35137414 PMC9115071

